# Multi-ancestry genome-wide association study and meta-analysis of lung function decline

**DOI:** 10.1186/s12931-026-03565-x

**Published:** 2026-02-28

**Authors:** Bonnie K. Patchen, Jingwen Zhang, Nathan Gaddis, Traci M. Bartz, Jing Chen, Catherine Debban, Hampton Leonard, Ngoc Quynh H. Nguyen, Jungkyun Seo, Courtney Tern, Richard Allen, Dawn L. DeMeo, Myriam Fornage, Carl Melbourne, Melyssa Minto, Matthew Moll, George T. O’Connor, Tess Pottinger, Bruce M. Psaty, Stephen S. Rich, Jerome I. Rotter, Edwin K. Silverman, Jeran Stratford, Chengyue Zhang, R. Graham Barr, Michael H. Cho, Sina A. Gharib, Ani Manichaikul, Kari North, Elizabeth C. Oelsner, Eleanor M. Simonsick, Martin D. Tobin, Bing Yu, Seung Hoan Choi, Josée Dupuis, Patricia A. Cassano, Dana B. Hancock

**Affiliations:** 1 Division of Nutritional Sciences, Cornell University, Ithaca, NY USA; 2 Channing Division of Network Medicine, Brigham and Women’s Hospital, Boston, MA USA; 3 Harvard Medical School, Boston, MA USA; 4 Boston University School of Public Health, Boston, MA USA; 5 RTI International, Research Triangle Park, NC USA; 6 Cardiovascular Health Research Unit, Departments of Biostatistics and Medicine, University of Washington, Seattle, WA USA; 7 Division of Public Health and Epidemiology, School of Medical Sciences, University of Leicester, Leicester, UK; 8 Department of Genome Sciences, University of Virginia School of Medicine, Charlottesville, VA USA; 9 Laboratory of Neurogenetics, National Institute of Aging, National Institute of Health, Bethesda, MD USA; 10 School of Public Health, University of Texas Health Science Center, Houston, TX USA; 11 Department of Epidemiology, University of North Carolina, Chapel Hill, NC USA; 12 Department of MetaBioHealth, Sungkyunkwan University (SKKU), Suwon, Republic of Korea; 13 Brown Foundation Institute of Molecular Medicine, McGovern Medical School, University of Texas Health Science Center, Houston, TX USA; 14 UK Biobank, Ltd, Stockport, UK; 15 Boston University School of Medicine, Boston, MA USA; 16 Institute for Genomic Medicine, Columbia University Irving Medical Center, New York, NY USA; 17 Cardiovascular Health Research Unit, Departments of Medicine, Epidemiology, Health Systems and Population Health, University of Washington, Seattle, WA USA; 18 The Institute for Translational Genomics and Population Sciences, Department of Pediatrics, The Lundquist Institute for Biomedical Innovation at Harbor-UCLA Medical Center, Torrance, CA USA; 19 Department of Medicine, Columbia University College of Physicians and Surgeons, New York, NY USA; 20 Division of Pulmonary, Critical Care and Sleep Medicine, University of Washington, Seattle, WA USA; 21 Intramural Research Program, National Institute on Aging, Baltimore, MD USA; 22 Department of Epidemiology, Biostatistics and Occupational Health, School of Population and Global Health, McGill University, Montréal, Québec Canada; 23 Department of Population Health Sciences, Division of Epidemiology, Weill Cornell Medicine, New York, NY USA

**Keywords:** Spirometry, Lung volume measurements, Genomics, Pulmonary disease, Chronic obstructive

## Abstract

**Background:**

Despite evidence for a genetic component, few genetic associations with lung function decline have been identified. We aimed to evaluate genome-wide associations and putative downstream functionality of genetic variants for lung function decline.

**Methods:**

We conducted genome-wide association study (GWAS) analyses of decline in FEV_1_, FVC, and FEV_1_/FVC in 52,056 White (*N* = 44,988), Black (*N* = 5,788), Hispanic (*N* = 550), and Chinese American (*N* = 730) participants across seven general population cohorts. GWAS analyses were stratified by cohort, ancestry, and sex. Results were combined in cross-ancestry and ancestry-specific meta-analyses. Significant variants available in two independent COPD-enriched cohorts were tested for replication.

**Results:**

We identified 361 distinct genome-wide significant (*p* < 5E-08) variants for one or more of the FEV_1_, FVC, and FEV_1_/FVC decline phenotypes, which overlapped with previously reported genetic signals for pulmonary traits. Four variants, or 10.3% of variants available for replication testing, were nominally associated (*p* < 0.05) with at least one decline phenotype in COPD-enriched cohorts. Gene-level analysis of GWAS results implicated 38 genes, many with consistent associations across ancestries or decline phenotypes. Annotation class analysis revealed enrichment of regulatory processes for corticosteroid biosynthesis and metabolism. Drug repurposing analysis identified 43 approved compounds targeting eight implicated genes.

**Conclusions:**

Our GWAS meta-analyses identified numerous genetic loci associated with lung function decline. These findings contribute knowledge to the genetic architecture of lung function decline, provide evidence for a role of corticosteroids in the etiology of lung function decline, and identify drug targets meriting further study for potential repurposing to slow lung function decline and mitigate lung disease.

**Supplementary Information:**

The online version contains supplementary material available at 10.1186/s12931-026-03565-x.

## Introduction

Lung function increases through childhood, plateaus in early adulthood, then declines with age. A faster or accelerated rate of decline in lung function is associated with chronic lung disease, including chronic obstructive pulmonary disease (COPD), a leading cause of death worldwide. Inflammation from smoking and other environmental exposures contributes to accelerated lung function decline and development of chronic lung disease, but not all smokers develop lung disease and non-smokers can develop chronic lung disease as well.

Genetic susceptibility also plays a role in lung function. Genome-wide association studies (GWAS) have identified hundreds of genetic loci associated with cross-sectional lung function, with heritability estimates ranging from 20% to 40% for forced expiratory volume in the first second (FEV_1_), forced vital capacity (FVC), and their ratio (FEV_1_/FVC) [1–3]. Heritability analyses suggest that there is also a genetic component to change in lung function over time, with estimates ranging from 5% to 18% across lung function phenotypes [4–6]. However, GWAS evaluating lung function decline are limited, and few genetic loci associated with lung function decline at genome-wide significance have been identified [5–10]. Larger GWAS with increased power are needed to identify robust genetic signals for lung function decline.

Here, we conducted the largest GWAS of lung function decline to date (total *N* = 52,056), included multiple ancestries, and achieved more coverage of the genome via newer imputation platforms to improve resolution of genetic signals for lung function decline. We identified novel variants associated with decline in FEV_1_, FVC, and FEV_1_/FVC at genome-wide significance, connected these variants to their corresponding genes and biologically relevant pathways, and identified drug targets that could be repurposed to slow decline and treat lung disease. These findings shed light on the genetic contribution to lung function decline.

## Methods

### Study populations and measurements

Our analyses included six US-based cohort studies from the Cohorts for Heart and Aging Research in Genomic Epidemiology (CHARGE) Consortium—Atherosclerosis Risk in Communities (ARIC), Coronary Artery Risk Development in Young Adults (CARDIA), Cardiovascular Health Study (CHS), Framingham Heart Study (FHS), Health, Aging and Body Composition Study (HABC), and Multi-Ethnic Study of Atherosclerosis (MESA)—and the UK Biobank. All studies included White/European ancestry (EA) participants, five included Black/African ancestry (AA) participants, and one included Hispanic and Chinese American participants.

FEV_1_ and FVC were measured by spirometry at baseline and follow-up visits following American Thoracic Society or European Respiratory Society recommendations current at time of assessment. FEV_1_ and FVC measurements passing quality control criteria for acceptability were included. The ratio of FEV_1_ to FVC (FEV_1_/FVC) was calculated when both FEV_1_ and FVC passed acceptability criteria. Pre-bronchodilator measures were used for all discovery studies.

Genotyping was performed as previously described [10]. Imputation was based on the Trans-Omics for Precision Medicine (CHARGE studies) or Haplotype Reference Consortium (UK Biobank) reference panels. Within each study variants with imputation values (R^2^ for CHARGE studies and info scores for UK Biobank) < 0.3 were excluded. Additional details on contributing cohorts, spirometry measurements, genotyping, and imputation methods are available in the online supplement.

### Statistical analysis

**Fig. 1:**
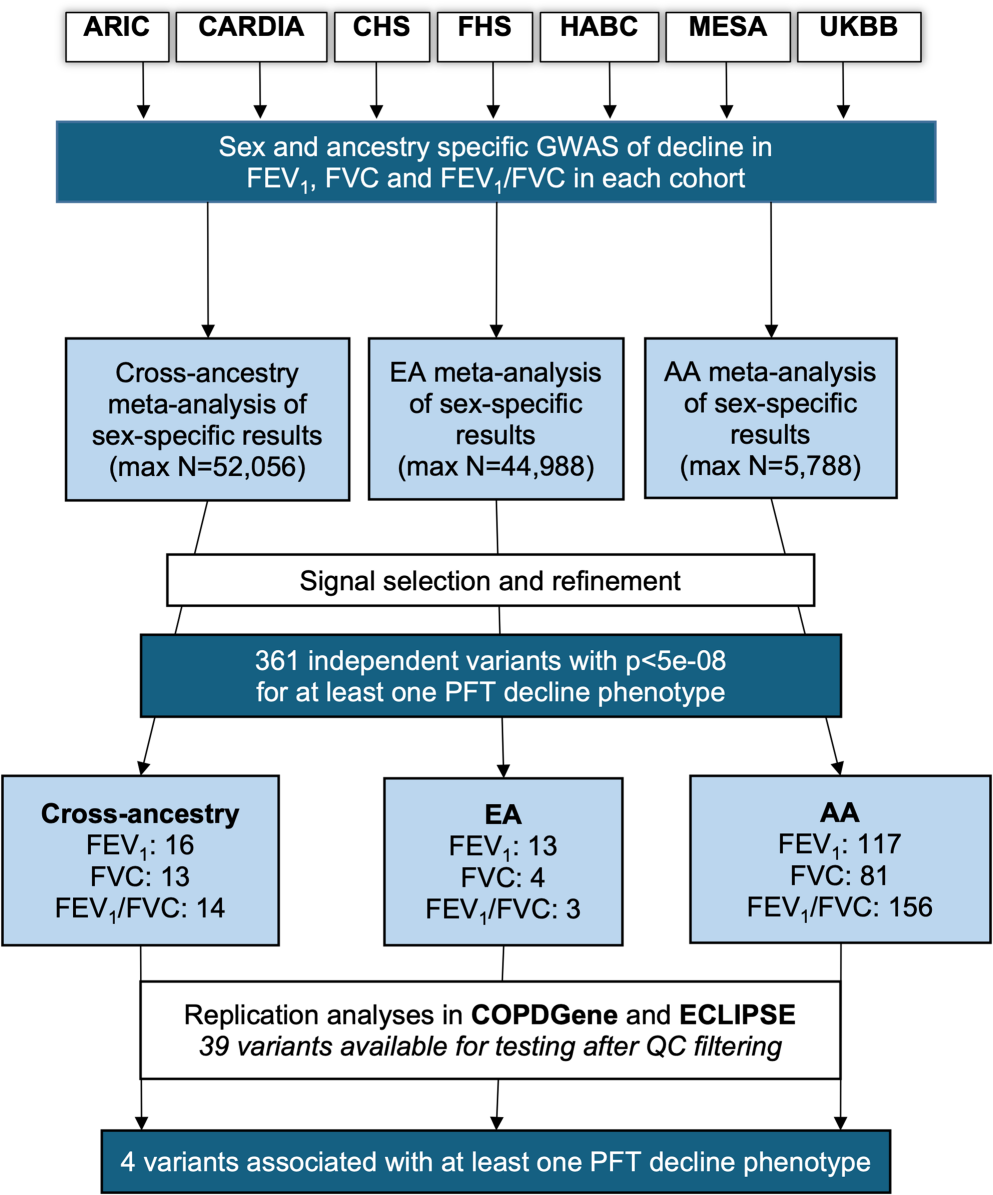
Study Design. Sex and ancestry-stratified GWAS analyses were performed separately in each cohort following a uniform analysis plan and summarized with inverse-variance weighted meta-analysis. Distinct variants with p-values passing genome-wide significance thresholds (p < 5E-08) were considered significant. Significant variants were tested for replication in two COPD-enriched cohorts following the discovery analysis plan. Replication was declared based on nominal significance of p<0.05

Figure 1 depicts the analysis scheme. We performed GWAS analyses for decline in FEV_1_, FVC, and FEV_1_/FVC separately in each cohort following a standardized analysis plan. We used the NHLBI Pooled Cohorts harmonized spirometry data where possible [11]. We estimated genome-wide variant associations with lung function decline using generalized estimating equations with robust standard error (GEE) and unstructured correlation structure. In all models, repeated measurements of FEV_1_, FVC, or FEV_1_/FVC were regressed on variant, elapsed time since first lung function measurement, and the variant × elapsed time multiplicative interaction term. We focused on the variant x time interaction term to identify variants associated with lung function decline. The GEE model was selected because it is robust for testing interaction terms in genetic association studies even when the interaction variable is mis-specified [12]. We stratified models by ancestry and sex and adjusted for key lung function covariates and genotype principal components. Details on cohort and ancestry specific genotype principal component adjustment are presented in the supplementary materials. Analyses were performed in R studio using the geepack R package [13]. To remove extreme values driven by small sample sizes, prior to meta-analysis, we filtered sex- and ancestry-specific results from each study to exclude variants present in < 30 participants, variants with minor allele counts < 20, and variants with effect sizes < − 50 or > 50 mL/year. Please see the online supplement for additional details.

We meta-analyzed the GWAS results with METAL using the inverse-variance based fixed effects method, which increases power and provides valid p-values under the null hypothesis. Heterogeneity of significant variants was evaluated with the I^2^ statistic, and for variants with substantial heterogeneity we also ran random-effects meta-analyses with GWAMA using the standard DerSimonian-Laird method [14]. Cross-ancestry analyses combined ancestry- and sex-specific results. Ancestry-specific meta-analyses were performed for EA and AA results; sample sizes were too small to conduct ancestry-specific meta-analyses for the other ancestry groups. We filtered meta-analysis results on total minor allele count < 200, variants present in < 25% of the maximum sample size, or variants present in fewer than two of the sex- and ancestry-specific cohort results.

### Signal selection

We processed the meta-analysis results using the Functional Mapping and Annotation (FUMA) SNP2GENE function with default parameters and a significance threshold of *p* < 5E-08 [15]. For the cross-ancestry and AA analyses we also report the number of variants significant at 2.5E-08 as more stringent significance thresholds may be needed for genomes with less linkage disequilibrium [16, 17]. Please see the online supplement for additional details.

### Replication of lung function decline-associated variants in COPD-enriched populations

We evaluated decline-associated variants in White (*N* = 4,778) and Black (*N* = 1,118) participants in two cohort studies enriched for COPD: the Genetic Epidemiology of COPD (COPDGene) [18, 19] and the Evaluation of COPD Longitudinally to Identify Predictive Surrogate End-points (ECLIPSE) [20]. Only variants passing QC filtering criteria with genomic position and alleles matching those in the discovery analyses were tested. Please see the online supplement for additional details.

### Heritability and genetic correlation with relevant pulmonary traits

We estimated heritability of lung function decline in the FHS cohort (*N* = 3,571 EA participants) using SOLAR. We evaluated decline phenotypes with evidence of significant heritability (*p* < 0.05) for genetic correlation with baseline FEV_1_ and FVC, baseline COPD, COPD at last observation, carbon monoxide diffusion capacity (DLCO), asthma, immunoglobulin E (IgE), emphysema, and interstitial lung abnormality (ILA). Please see the online supplement for further details.

### Gene-based tests and functional validation analyses

We performed gene-based tests for meta-analysis results using MAGMA. We evaluated gene expression in lung tissue using the GTEx platform [21]. We performed co-localization analysis for blood and lung expression quantitative trait loci (eQTLs) using TOPMed cross-cohort multi-ancestry cis-eQTL data and the R package coloc [22, 23]. Genetically-predicted protein level associations with decline phenotypes were assessed using the S-PrediXcan framework with protein prediction models based on ancestry-specific pQTLs derived from European and African ancestry individuals in the ARIC cohort [24–26]. We evaluated gene-set enrichment with the FUMA GENE2FUNC function [15]. We explored pathway and disease enrichment patterns with the Genomic Regions Enrichment of Annotations Tool (GREAT) [27, 28]. Please see the online supplement for additional details.

### Overlap with previously reported signals

We evaluated decline-associated variants and genes for associations with relevant spirometry or lung disease phenotypes in the GWAS catalogue and Phenoscanner databases [29–31]. We considered the following traits: cross-sectional lung function (spirometry), COPD, emphysema/chronic bronchitis, asthma/allergic disease, other respiratory disease and death from respiratory disease. For variants we used a ± 500 kb window for queries rather than an LD threshold because the majority of variants were not in the 1000 Genomes reference panel and had limited information on LD. For genes we considered all variants annotated to genes significant in the MAGMA analysis. All queries were performed in R studio 3.4 using the LDlinkR and Phenoscanner R packages [30, 32]. 

### Drug repurposing analyses

Drug repurposing analyses focused on protein coding genes significant in the MAGMA analysis. We identified existing pharmacotherapies targeting these genes by querying four large drug-related resources, including Pharos [33, 34], Open Targets [35], Therapeutic Target Database [36], and DrugBank [37]. 

## Results

### Participants

Our analyses included 52,056 self-reported White (*N* = 44,988), Black (*N* = 5788), Hispanic (*N* = 550), and Chinese American (*N* = 730) participants (Fig. 1; Table 1). 58% were never smokers, 32% were former smokers, and 10% were current smokers. Participants were followed for a mean of 8.6 years with a mean of 2.3 spirometry measurements over the follow-up period, with cohort- and race and ethnicity-specific means ranging from 4.2 to 18.7 and 1.7‒4.6, respectively. Cohort-specific characteristics are presented in the online supplement (Table S1).

**Table 1:** Participant characteristics

*N*	Total	European ancestry	African ancestry	Hispanic	Chinese ancestry
	52,056	44,988	5,788	730	550
Cohort, N (%)					
ARIC	11,091 (21.3)	8,704 (19.3)	2,387 (41.2)	—	—
CARDIA	2,550 (4.9)	1,611 (3.6)	939 (16.2)	—	—
CHS	3,663 (7.0)	3,085 (6.9)	578 (10.0)	—	—
FHS	2,013 (3.9)	2,013 (4.5)	—	—	—
HABC	2,464 (4.7)	1,505 (3.3)	959 (16.6)	730	—
MESA	3,876 (7.4)	1,671 (3.7)	925 (16.0)	(100.0)	550 (100.0)
UKBB	26,399 (50.7)	26,399 (58.7)	—	—	—
Female, N (%)	28,455 (54.6)	24,718 (54.9)	3,348 (57.8)	389 (53.5)	270 (49.1)
Age, yr	56.5 (11.9)	56.2 (10.9)	56.5 (17.5)	66.4 (10.3)	66.0 (9.7)
Height, cm	168.3 (9.3)	168.6 (9.2)	167.3 (9.3)	161.3 (9.2)	161.5 (8.7)
Weight, kg	76.4 (15.7)	76.0 (15.4)	80.6 (17.4)	77.2 (15.7)	63.0 (11.0)
Current smoking, N (%)	5,477 (10.5)	4,180 (9.3)	1,128 (19.5)	58 (7.9)	24 (4.4)
Former smoking, N (%)	16,629 (31.9)	14,315 (31.8)	1,843 (31.8)	330 (45.2)	141 (25.6)
Baseline FEV_1_, mL	2,789 (814)	2,860 (799)	2,351 (773)	2,328 (720)	2,189 (653)
Baseline FVC, mL	3,696 (1016)	3,798 (994)	3,060 (916)	3,059 (897)	2,915 (841)
Baseline FEV_1_/FVC, %	75.4 (7.5)	75.2 (7.2)	76.7 (9.0)	76.1 (8.0)	75.2 (7.4)
N PFT measurements	2.3 (1.0)	2.3 (0.9)	2.3 (1.9)	1.7 (0.8)	1.8 (0.8)
Total follow-up, years	8.6 (6.3)	8.7 (6.0)	8.3 (8.2)	4.2 (4.9)	5.5 (5.2)
Time between pulmonary function test visits, years	7.3 (5.2)	7.1 (5.0)	8.0 (7.1)	7.6 (3.2)	8.2 (3.2)

Values are presented as mean (SD) unless otherwise specified

### GWAS meta-analyses identify robust associations with lung function decline

Our analyses captured up to 11.8 million genotyped and imputed variants. Genomic inflation values (λ_gc_) across cohorts ranged from 0.57 to 1.30 after QC filtering. Cohort results with λ_gc_ > 1 were adjusted using the genomic control function in METAL. We found little evidence for genomic inflation after meta-analysis (cross ancestry λ_gc_ = 1.03 for FEV_1_ and 1.02 for both FVC and FEV_1_/FVC; EA λ_gc_ = 1.03 for FEV_1_, 1.01 for FVC and 1.02 for FVC; AA λ_gc_ = 1.08 for FEV_1_, 1.05 for FVC and 1.03 for FEV_1_/FVC) (Figure S1).

**Fig. 2:**
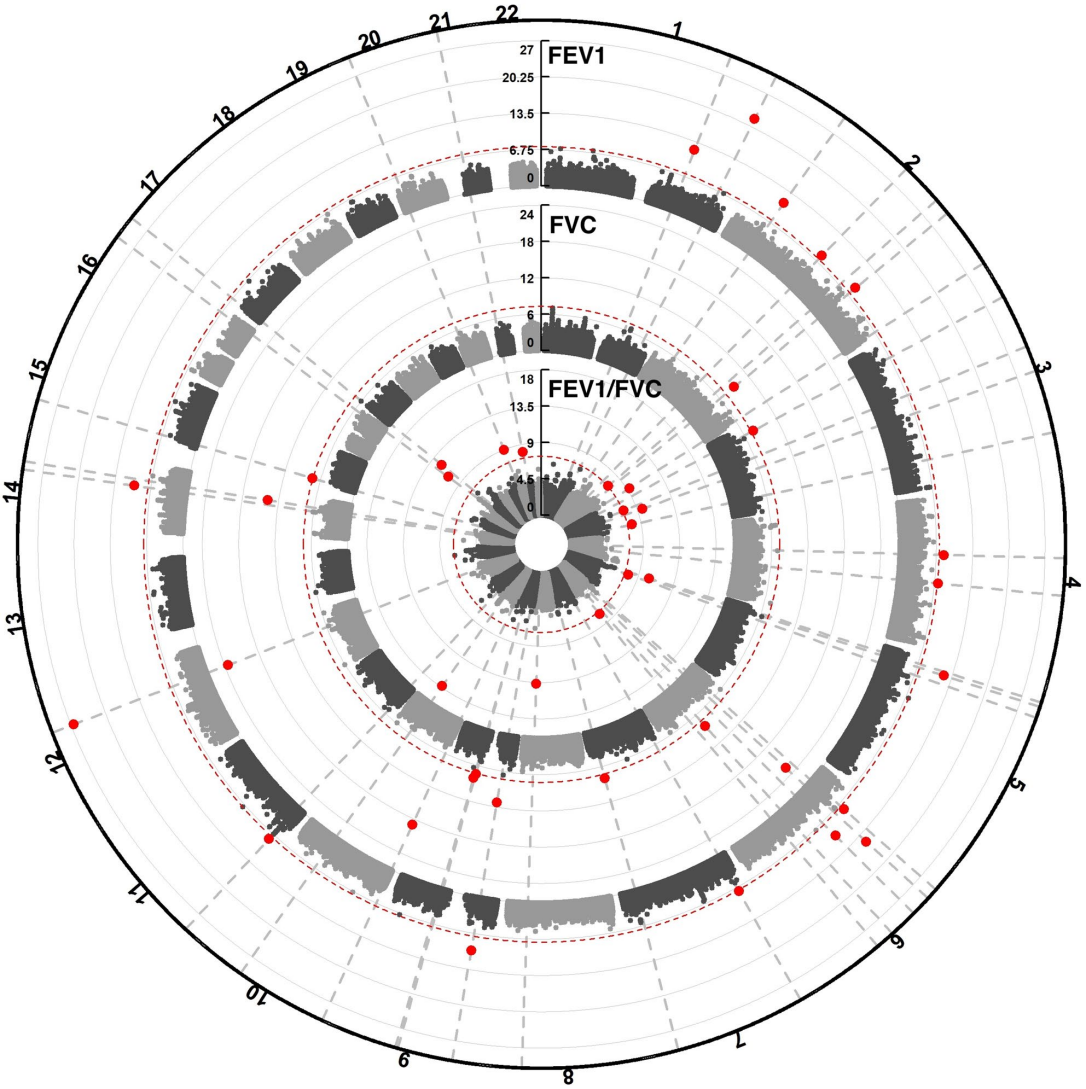
Circular Manhattan Plot of Cross-Ancestry Results. Genome-wide results for decline in FEV_1_ (outer circle), FVC (middle circle), and FEV_1_/FVC (inner circle) from the cross-ancestry analyses. Dotted red lines denotes the genome-wide significance threshold of p = 5E-08. Red circles represent variants passing genome-wide significance

Cross-ancestry meta-analyses identified 39 distinct variants (R^2^ < 0.1) associated with lung function decline at *p* < 5E-08 (Fig. 2, Table S2). Twelve variants were for FEV_1_, nine were for FVC, four were for both FEV_1_ and FVC, and 14 were for FEV_1_/FVC. These variants represent novel signals for longitudinal decline in lung function. Most of the variants (36 of 39) had minor allele frequencies < 0.05 in the study population (Figure S2). 32 variants were significant at the more stringent threshold of *p* < 2.5E-08 [17].

Ancestry-specific meta-analyses identified 20 distinct variants for EA (13 for FEV_1_, four for FVC, and three for FEV_1_/FVC; Figure S3) and 307 distinct variants for AA (79 for FEV_1_, 45 for FVC, and 138 for FEV_1_/FVC, 9 for both FEV_1_ and FEV_1_/FVC, 7 for both FVC and FEV_1_/FVC, and two for all three phenotypes; Figure S4). All variants significant in the EA analyses had minor allele frequencies < 0.05, whereas most variants significant in the AA analyses (224 of 307) had minor allele frequencies ≥ 0.05 (Figure S2). 277 variants identified in the AA analyses were significant at *p* < 2.5E-08 [17]. Notably, a majority (87%) of variants identified in the AA analyses were present (i.e. passing our QC filtering criteria for MAC) only in the ARIC cohort, which we attribute to ARIC having largest AA sample size (*N* = 2,387, ~ 41% of the total AA sample).

Eleven variants significant in the cross-ancestry meta-analyses were also significant or within 500 kb of variants significant in the EA- or AA-specific meta-analyses (Table 2). One variant significant only in the AA analyses, rs32137 in the *CTNND2* gene on chromosome 5, is within ± 500 kb of a variant previously reported for nominal association (*p* = 6E-06) with FEV_1_ decline in a GWAS of asthmatics [8]. None of the other variants have previously been associated with lung function decline.

**Table 2:** Cross-ancestry variants significant or within 500 kb of variants significant in the European or African ancestry analyses

rsID	Chromosome: position (hg38)	Reference/ alternate alleles	Closest Gene	*N Studies	*Total Sample size	*MAF	*Phenotype	*Effect Estimate	*P-value	Het I^2^
rs114110050	1:181557627	G/C	*CACNA1E*	9	41,303	0.02	FEV_1_	−6.07	1.0E-12	88
rs561006775	2:43650017	AG/A	*PLEKHH*2	7	15,231	0.20	FEV_1_	7.48	6.3E-12	86
rs139399765	3:20815668	C/T	*RNU6-815P*	7	39,318	0.04	FVC	−4.82	2.2E-08	88
rs149963074	3:99726132	C/T	*COL8A1*	9	16,903	0.02	RATIO	0.13	2.1E-10	89
rs73875952	3:161893323	G/C	*RP11-774I5.1*	6	14,219	0.01	RATIO	−0.10	1.4E-08	84
rs111351387	4:71826274	G/T	*RP11-545L5.1*	14	20,927	0.06	FEV_1_	3.83	6.3E-09	56
rs559126463	6:55623623	G/GATATATAC	*HMGCLL1*	11	18,621	0.04	FEV_1_	4.64	5.2E-11	90
rs78349759	6:83786677	C/T	*RP3-393K13.1*	9	41,348	0.01	FVC	5.64	4.2E-09	47
rs150709492	7:8801178	C/T	*NXPH1*	15	46,421	0.02	FEV_1_	−3.81	2.9E-08	86
rs2921778	8:116877202	G/T	*RAD21-AS1*	13	16,627	0.03	RATIO	0.10	2.2E-14	84
rs540140520	9:78892132	AC/A	*KRT18P24*	6	27,725	0.01	FVC	−6.05	6.7E-09	87

*N studies, total sample size, alternate allele frequency, decline phenotype, effect estimate, p-value and het I^2^ are from cross-ancestry fixed effects analysis

There was substantial heterogeneity in both the cross-ancestry and ancestry-specific results, with median I^2^ values of 84%, 55% and 54% for the cross-ancestry, EA and AA results respectively, and a total of 236 PFT association results (from 218 variants) having I^2^ values > 50%. Of these, two AA results, rs78649925 for FEV_1_ and rs17151153 for FEV_1_/FVC were significant at *p* < 5.0E-08 in random-effects meta-analysis. I^2^ values and random-effects p-values for all results significant in the fixed-effects meta-analyses are presented in Table S2.

### Genetic signals for lung function decline suggest replicability in independent COPD-enriched populations

A limited set of 39 of the 361 distinct variants identified in our primary analysis were available (passing QC criteria and aligned for genomic position and reference/alternative alleles) for replication testing in COPDGene and/or ECLIPSE. This included 28 variants for the EA meta-analysis, 12 variants for the AA meta-analysis and 4 variants for the cross-ancestry meta-analysis. No variants surpassed Bonferroni correction (*p* < 0.0013 [*p* < 0.05/39 variants)]). Two variants were nominally significant (*p* < 0.05) with a consistent direction of effect for at least one decline phenotype (Table 3). Results were similar in models adjusted for COPD status and disease stage (correlation coefficient [corr] = 0.90, 95% CI = 0.86 to 0.93), with two additional variant—phenotype associations reaching nominal significance (Table 3). Only one variant, rs57862396 on chromosome 9, replicated for the same phenotype and direction of effect. For this variant, the T (alternate) allele was associated with a slower rate or attenuation in FEV1/FVC decline of 0.48% per year in the discovery cohorts (*p* = 3.94E-08) and 0.13% per year in the replication cohorts (*p* = 0.011). The other nominally significant associations were for different phenotypes between the discovery and replication analyses. P-values for all variants tested for replication are presented in Table S4.

**Table 3:** Decline variants with nominal evidence (*p* < 0.05) for decline associations in COPD-enriched cohorts

rsID	Chromosome: position	Association in Discovery Cohorts							Association in Replication Cohorts						
		Ancestry	Pheno	EA	EAF	Effect mL or % per year	*P*-value	Het I^2^	Ancestry	Pheno	EA	EAF	Effect mL or % per year	*P*-value	Het I^2^
*Associations seen in primary replication analysis*															
rs57862396	9:33245969	AA	RATIO	T	0.05	0.48	3.9E-08	70	EA	RATIO	T	0.03	0.13	0.0112	0
rs6814777	4:142766606	AA	RATIO	A	0.85	0.37	2.7E-14	88	EA	FVC	A	0.97	37.5	0.0326	37
*Additional associations seen in replication analysis adjusting for COPD status and disease stage*															
rs12445073	16:86705263	AA	RATIO	T	0.09	0.19	1.3E-08	0	EA	FVC	T	0.04	41.9	0.0272	0
rs13391412	2:116850979	AA	FVC	T	0.04	-20.8	2.8E-08	0	EA	RATIO	T	0.02	-0.21	0.0289	0

*Pheno* Pulmonary function decline phenotype, *EA* Effect allele, *EAF* Effect allele frequency

### Genetic signals for lung function decline overlap with signals for relevant pulmonary phenotypes

**Fig. 3:**
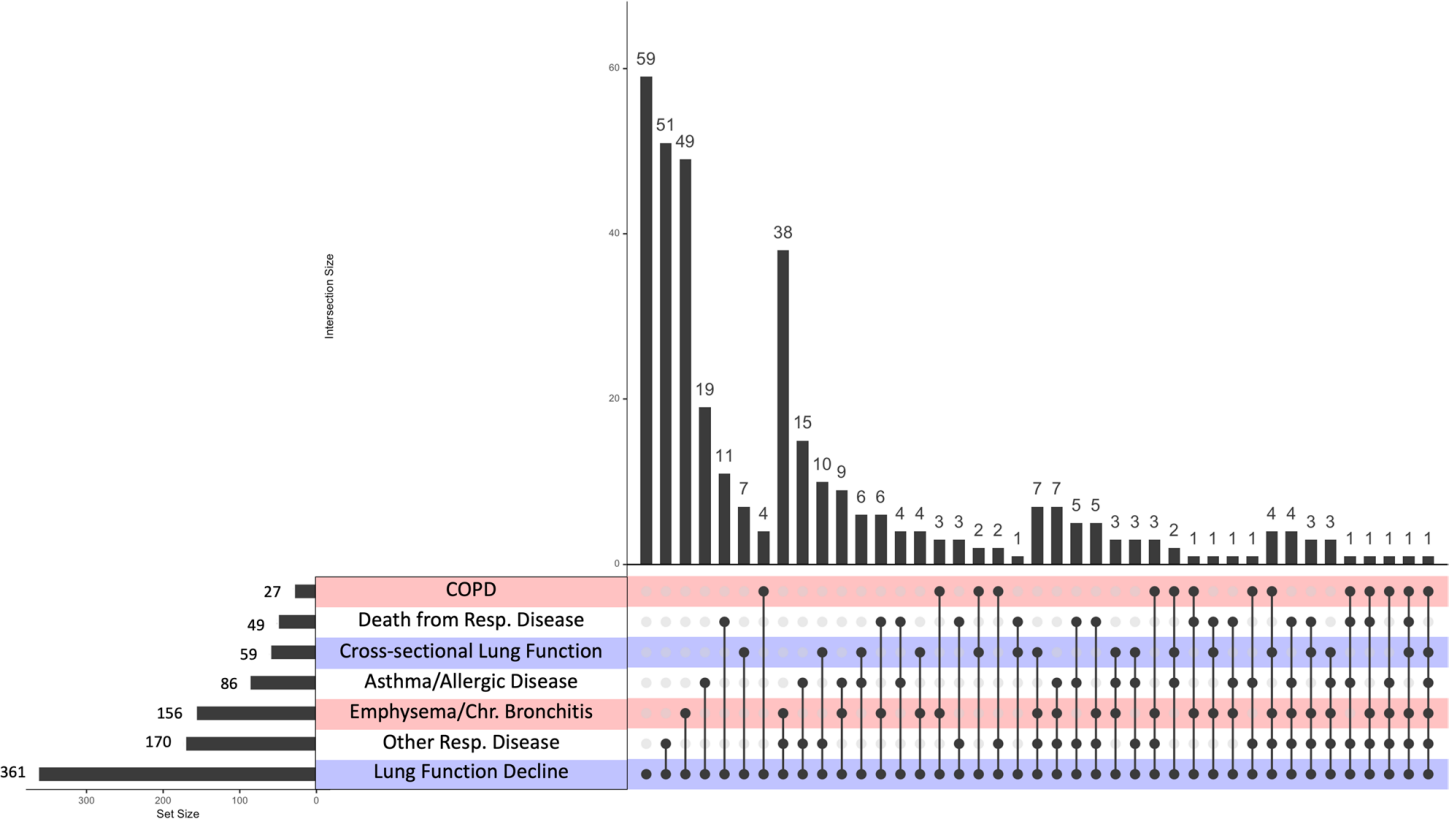
Upset plot showing overlap of lung function decline-associated variants with previously reported signals for relevant pulmonary phenotypes. Significant decline-associated variants were evaluated for prior associations with relevant pulmonary traits (COPD, death from respiratory disease, cross-sectional lung function, asthma/allergic disease, emphysema/chronic bronchitis, and other/non-specified respiratory disease). Dots connected by lines depict intersecting sets and vertical bars show the number of variants in each intersection. The first column shows the variants identified in this GWAS that were not previously reported for the pulmonary traits evaluated

Of the 361 distinct variants (R^2^ < 0.1) identified in cross-ancestry or ancestry-specific analyses, 302 were ± 500 kb of variants previously reported for associations with other pulmonary phenotypes, including cross-sectional lung function (58 variants); COPD (27 variants); asthma/allergic disease (85 variants); emphysema or chronic bronchitis (155 variants); pulmonary fibrosis, interstitial lung disease, or other unspecified respiratory disease (169 variants); and death from respiratory disease (48 variants) (Fig. 3, Tables S5 and S6). These variant sets overlapped: 103 decline variants were ± 500 kb of variants associated with two traits; 39 were ± 500 kb of variants associated with three traits; 17 were ± 500 kb of variants associated with four traits; and two variants, rs569142166 in the *RAB31* gene on chromosome 18 and rs561379073 near the *LINCO1139* gene on chromosome 1, were ± 500 kb of variants associated with five of the six traits evaluated (Fig. 3, Tables S5 and S6). Additionally, several variants previously reported for genome-wide significant associations with cross-sectional spirometry measures (164 variants), COPD (63 variants), asthma (113 variants), and emphysema (2 variants) showed nominal evidence (*p* < 0.05) for associations with lung function decline phenotypes in our analyses (Table S7). We were unable to fully assess LD of our decline-associated variants with previously identified variants as most variants significant in our study were not present in the 1000 Genomes reference panel.

### Lung function decline is heritable and genetically correlated with relevant pulmonary traits

We estimated the heritability of lung function decline in the FHS cohort at 0.04 (*p* = 0.180) for FEV_1_ decline, 0.06 (*p* = 0.124) for FVC decline, and 0.15 (*p* = 0.001) for FEV_1_/FVC decline. Genetic correlation with relevant pulmonary traits was estimated only for decline in FEV_1_/FVC because it was the only decline phenotype with a statistically significant heritability estimate. These analyses suggested positive correlations of FEV_1_/FVC decline with baseline FVC (corr = 0.22, se = 0.11, *p* = 0.051) and DLCO (corr = 0.42, se = 0.20, *p* = 0.052) and negative correlations with COPD at last study visit (corr=-0.58, se = 0.24, *p* = 0.070) and ILA (corr=-0.50, se = 0.30, *p* = 0.065) (Table S8).

### Colocalization of decline-associated variants with blood and lung eQTL

**Fig. 4:**
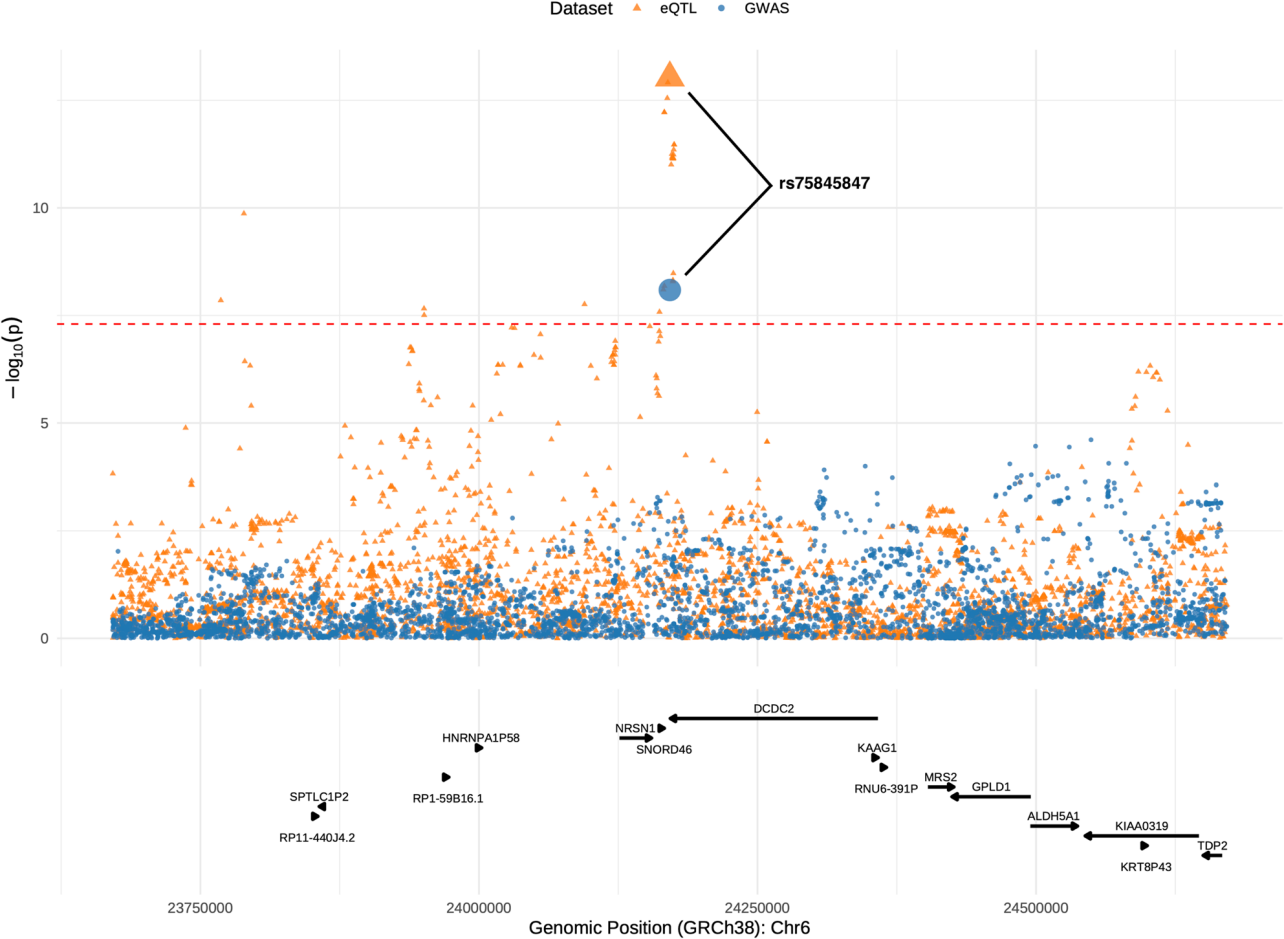
Locus plot of GWAS decline variant with strong evidence of eQTL colocalization. Locus plot overlaying our cross-ancestry FEV_1_ decline GWAS results (blue circles) with the TOPMed blood eQTL results (orange triangles) for the +/-500 kb around rs75845847 on chromosome 6 near the DCDC2 gene. The variant (rs75845847) with the largest SNP-level posterior probability of being the shared causal variant is enlarged. GRCh38 positions are displayed

We queried regions of +/-500 kb around the 361 decline-associated variants identified in our GWAS analyses and found one region with strong evidence of eQTL colocalization (posterior probability of a shared causal variant [ppH4] > 0.8) and three regions with moderate evidence for colocalization (ppH4 > 0.5) (Table S9). Further evaluation of variant-level results and locus plots confirmed colocalization of rs75845847, a 500B downstream variant of the *DCDC2* gene on chromosome 6 that was associated with FEV_1_ decline in our cross-ancestry analyses, with a blood cis-eQTL (variant-level ppH4 = 0.99, Fig. 4, Table S10). For the other regions, the variant-level results and locus plots suggest that the variants implicated for eQTL colocalization (variant-level ppH4 > 0.5) represent signals distinct from those identified in our GWAS, as these were > 450 kb from the decline-associated variants with little evidence of LD (Figure S5, Table S10).

### Genetically-predicted protein level associations with decline phenotypes

We identified one predicted protein-decline association that was significant at FDR < 0.1: KLB with FEV_1_ decline in the AA specific analyses (Table S11). KLB levels were also associated at nominal significance (*p* < 0.05) with decline in FEV_1_ in the AA analyses, with consistent directions of effect across the EA and cross-ancestry analyses (Figure S6). Results for all nominally significant (*p* < 0.05) protein—decline associations (520 associations for 299 unique proteins) are presented in table S11.

### Lung function decline associated-variants implicate 38 genes

**Fig. 5:**
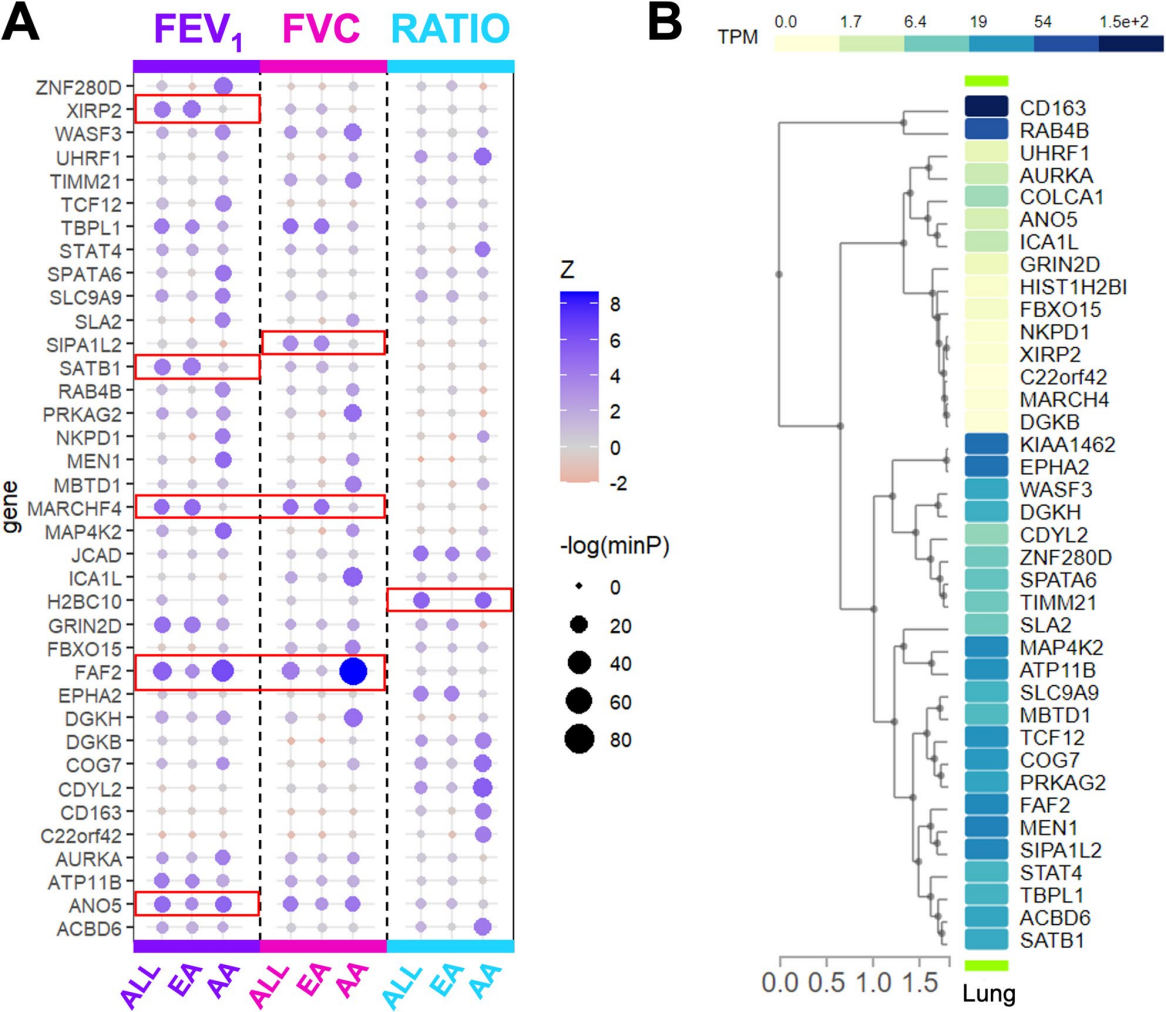
Gene-based testing of cross-ancestry and ancestry-specific GWAS results. Genes implicated from gene analysis of our GWAS results. **A** Z-score comparison across ancestries and decline phenotypes. Color corresponds to Z score value and circle size corresponds to p-value. Red boxes highlight genes with consistent and significant associations across ancestries or phenotypes. **B** GTEx data for lung tissue. Note: KIAA1426 synonymous for JCAD; HIST1H2BI synonymous for H2BC10

Gene-based testing of GWAS results implicated 38 genes significant for at least one decline phenotype (Table S12). Eight genes, including *XIRP2*, *GRIN2D*, *SATB1*, *MARCHF4*, *SIPA1L2*, *ANO5*, *H2BC10*, and *FAF2*, were consistent across ancestries or phenotypes (Fig. 5A). GTEx evaluation confirmed expression in lung tissue (Fig. 5B). Twenty-eight genes were previously reported for GWAS associations with relevant pulmonary traits (Table S13). Ten genes, including *FAF2*, *MARCHF4*, *GRIN2D*, *AURKA*, *MAP4K2*, *H2BC10*, *COG7*, *MEN1*, *SLA2*, and *CD163*, were not previously reported for lung disease–related traits. Only one gene implicated in the gene-based testing, *EPHA2*, was represented in the protein prediction models underlying the S-PrediXcan analyses. Predicted protein levels of EPHA2 showed a nominally-significant (*p* < 0.05) association with FEV1/FVC decline in the AA S-PrediXcan analyses (Table S11, Figure S6).

### Lung function decline-associated genes are enriched for positional and other functional gene sets

Gene set enrichment results are shown in the online supplement (Table S14). Cross-ancestry results were enriched for positional gene sets at chr6p21 and chr3q12 and the GWAS catalogue gene-set for height. EA results were enriched for positional gene sets at chr12q12, chr3p24, and chr7p14. AA results were enriched for 43 positional gene sets, 16 GWAS catalogue gene sets (including COPD and idiopathic pulmonary fibrosis), two cancer/computational gene sets, two chemical and genetic perturbation/curated gene sets, two micro-RNA targets gene sets, and one immunologic signature.

### Lung function decline-associated genes are enriched for biological pathways

**Fig. 6:**
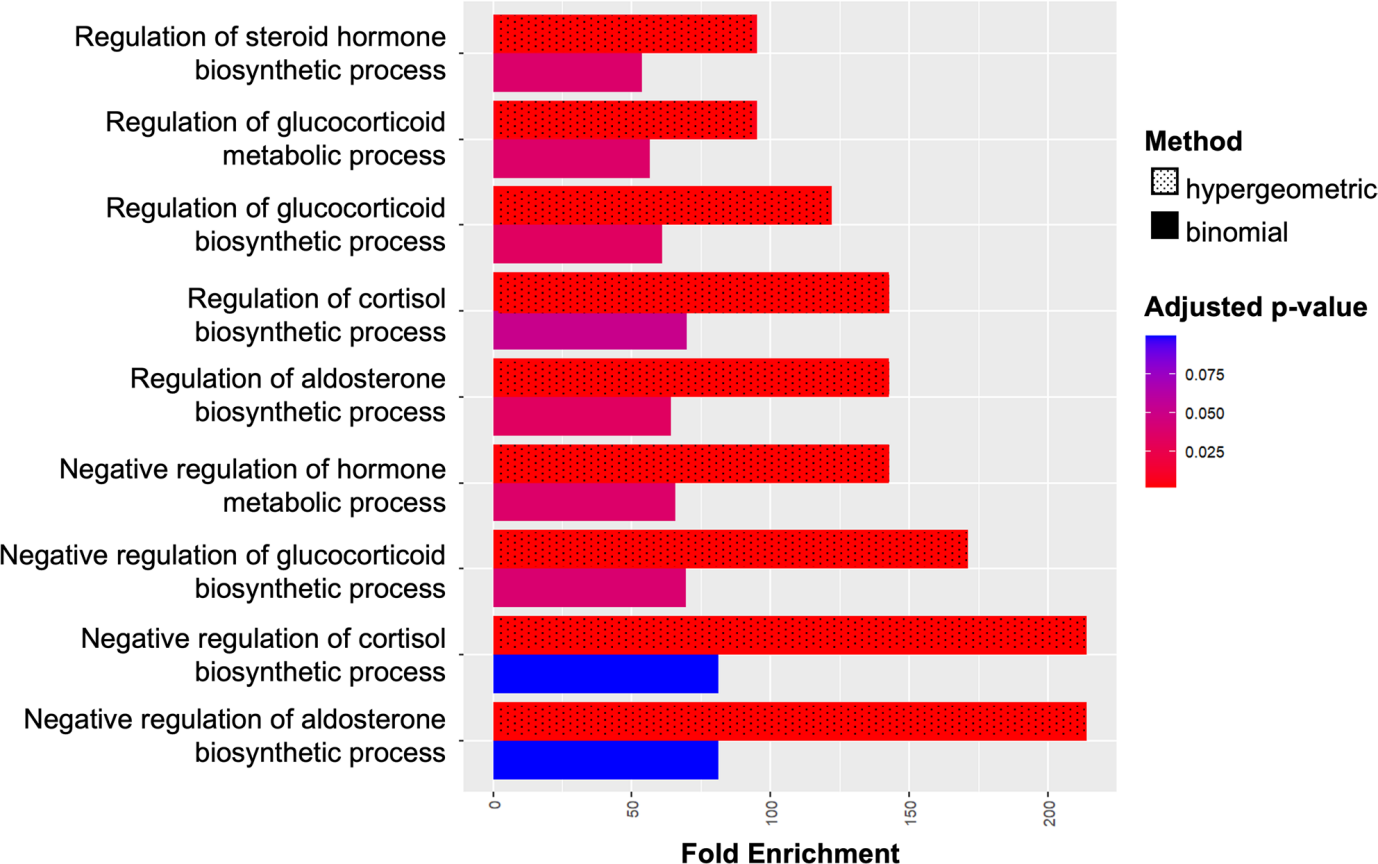
Enrichment of processes regulating glucocorticoid and mineralocorticoid biosynthesis. Annotation class analysis of GWAS results revealed significant enrichment of Gene Ontology (GO) biological processes for corticosteroid regulatory pathways. Fold enrichment calculated using the binomial (textured) and hypergeometric (untextured) methods are shown. Color represents false discovery rate adjusted p values

FUMA-defined genomic loci for the cross-ancestry analysis showed significant enrichment (false discovery rate–adjusted *p* < 0.05) of Gene Ontology biological processes for the regulation of glucocorticoid and mineralocorticoid biosynthesis (Fig. 6). There was no further evidence for pathway or disease enrichment for the ancestry-specific analyses.

### Lung function decline-associated genes represent druggable targets

Most genes (37/38) from the MAGMA analysis were protein coding genes. Queries across four drug repurposing databases identified 43 approved compounds targeting eight genes (*AURKA*, *CD163*, *DGKB*, *DGKH*, *EPHA2*, *GRIN2D*, *MAP4K2*, *PRKAG2*). The full set of identified compounds is available in Table S15. Inspection of these results highlighted the N-methyl-D-aspartate (NMDA) receptor GRIN2D and the receptor tyrosine kinase EphA2, which had 32 and 6 compounds, respectively. The remaining genes were targeted by one to three compounds each.

## Discussion

In these multi-ancestry GWAS meta-analyses of lung function decline, we identified 361 novel genetic variants associated with lung function decline at genome-wide significance in general population cohorts, with strong evidence for eQTL colocalization for one variant. The suggestive evidence that we observed of replication for some variants in COPD-enriched study populations and pleiotropy with other pulmonary phenotypes merit additional investigation with larger sample sizes. Gene- and protein-level analyses highlighted several genes with consistent associations across decline phenotypes or ancestries, including some that overlapped with genes previously reported for cross-sectional lung function or other pulmonary traits and some that were (to our knowledge) novel. Pathway analyses revealed enrichment for regulatory processes for corticosteroid biosynthesis and metabolism. These findings contribute to our understanding of the genetic architecture of lung function decline and highlight a potential role of corticosteroid metabolism in the etiology of lung function decline.

The largest prior GWAS meta-analysis included over 27,000 EA participants and did not identify any genome-wide significant variants in the overall meta-analysis but identified one variant, rs507211 in *ME3*, associated with FEV_1_ decline in analyses limited to studies with three or more measurements [10]. Two other GWAS, both in ~ 6,500 Korean participants with three or more repeated measurements, each found one variant passing genome-wide significance [5, 6]: These included rs2445936 near *CEP164* associated with FEV_1_/FVC decline and rs2272402 in *SLC6A1* associated with FEV_1_ and FVC decline. Genome-wide significant findings for lung function decline in lung disease cohorts are similarly sparse, and generally not validated in general population cohorts [29–31]. The current study combined multiple ancestries from general population cohorts and leveraged newer imputation panels, increasing both sample size and genomic coverage. Our discovery of 361 genome-wide significant variants greatly expands the number of loci associated with longitudinal lung function. One variant identified in our AA analyses, rs32137 in *CTNND2*, was reported as nominally significant in a prior GWAS of lung function decline in asthmatics [8]. The rest, to our knowledge, represent novel signals for lung function decline.

Lung function decline contributes to cross-sectional lung function, and chronic lung diseases are characterized by impaired lung function and accelerated lung function decline [38–43]. The largest GWAS of cross-sectional lung function demonstrated strong associations of polygenic risk scores for impaired lung function with multiple chronic lung diseases, suggesting an overlap in underlying genetic architecture [2]. In the present study, we also found evidence supporting genetic overlap between lung function decline and other pulmonary traits, including cross-sectional lung function, COPD, asthma, emphysema, chronic bronchitis, pulmonary fibrosis, and interstitial lung disease. We also found suggestive evidence of a genetic correlation between FEV_1_/FVC decline and baseline FVC, COPD, carbon monoxide diffusion capacity, and interstitial lung abnormality in a subset of EA participants from one cohort (FHS) for which we had access to individual-level data. Given the limited sample for this analysis, these results may be strengthened by using individual-level data from larger, more diverse populations. Further research to understand the shared genetics of lung function decline and other pulmonary traits is warranted.

Pathway analyses of our findings highlighted regulatory processes for corticosteroid biosynthesis and metabolism. Corticosteroids, especially glucocorticoids, have potent anti-inflammatory and immunomodulatory effects, and synthetic versions are widely used for management of inflammatory and autoimmune diseases, including chronic lung diseases [44, 45]. Endogenous glucocorticoids play a role in lung maturation, which in turn impacts longitudinal lung function trajectories [45]. Endogenous cortisol, the predominant glucocorticoid, has been associated with accelerated lung function decline and the risk and severity of asthma, COPD, and chronic nonspecific lung disease [46–52]. Our findings contribute to the evidence supporting a role of corticosteroids in lung health.

Many of the genes and proteins implicated by our GWAS findings have not been previously associated with lung function. However, our drug repurposing analysis based on these genes identified compounds associated with known lung health modulators, such as the antioxidant N-acetylcysteine (NAC) that has been evaluated in COPD pathogenesis and progression [53] and the expectorant guaifenesin that is used to thin mucus and relieve congestion in chronic lung disease [54], increasing our confidence that these findings could translate to actionable targets. This convergence of established and novel findings strengthens the foundation for future research endeavors [53]. 

The identification of the *GRIN2D* gene, which encodes an NMDA receptor, is of particular interest, as emerging evidence is demonstrating the importance of NMDA receptors in lung function. Studies have demonstrated that NMDA receptors are present in human lung tissue and lead to calcium release and airway contraction [55]. Furthermore, NMDA receptor modulation has been proposed as part of a multi-pronged approach to improve airway smooth muscle function in Severe Acute Respiratory Syndrome patients [56, 57]. *GRIN2D*, which showed consistent associations across ancestries, is targeted by 32 approved compounds and represents a promising drug repurposing target. Further investigation into these compounds and *GRIN2D* is needed to elucidate specific mechanisms and therapeutic potential.

We observed in protein prediction models that beta klotho (KLB), a co-receptor of fibroblast growth factors (FGF) 19 and 21 that plays a role in carbohydrate and lipid metabolism, is predicted to be associated with FEV_1_ decline. While little is known about the association of KLB with lung function, recent evidence links KLB and FGF21 to pulmonary fibrosis, suggesting KLB/FGF signaling may have an anti-fibrotic effect in the lungs [58, 59]. Interestingly, the paralogous gene alpha klotho (KL), an established anti-aging gene that is a co-receptor of FGF23, has recently been associated with cross-sectional lung function, COPD, IPF and cigarette smoking [60–64]. We observed a nominal association (*p* < 0.05) of KL with decline in FEV1 and FEV1/FVC in our protein prediction analyses (Table S11). Further research on the klotho genes and FGF signaling in the context of lung function decline and chronic lung disease is warranted.

This study has several limitations. First, to increase power for discovery, we combined all contributing general population cohorts rather than reserving cohorts for replication. To mitigate this and also to increase clinical relevance of the findings, we tested for replication in two cohorts enriched for COPD. However many of the decline-associated variants had low minor allele frequencies (MAF < 0.05), and while most had high imputation quality (imputation value > 0.9), increasing our confidence in these signals, our ability to replicate was limited by the smaller sample sizes of the replication cohorts, meaning that a majority of the decline-associated variants did not pass our QC criteria for replication analyses. These analyses were also limited to EA and AA participants; further replication in larger, more diverse populations is needed. Second, many variants identified in the AA analyses only passed our QC criteria in one cohort. Although this cohort represented nearly half the total AA sample, further investigation of these variants in cohorts with larger AA sample sizes is needed. Third, our multi-ancestry analyses included participants identifying as White, Black, Asian, or Hispanic, analyzed separately and combined through meta-analysis. Although multiple cohorts included both White and Black participants, only one cohort included Asian and Hispanic participants and sample sizes were too small to perform ancestry-specific analyses for these participant groups. Fourth, the length of follow-up and number of repeated measures varied across cohorts, which could contribute to heterogeneity in the precision of variant–decline association estimates. Fifth, we primarily used fixed-effects meta-analyses assuming one true underlying effect. However, for many variants, there was substantial heterogeneity despite covariate adjustment, and most variants did not pass genome-wide significance thresholds in random-effects meta-analysis. Lastly, although we accounted for key covariates for lung function and its decline, most notably by stratifying by sex and adjusting for smoking variables, we did not evaluate interactions of these covariates with our findings. Sex-specific and gene-by-smoking interaction analyses could shed further light on genetics underlying differences in lung function decline by these factors.

In summary, in the first multi-ancestry and largest GWAS of lung function decline to date, we identified numerous genome-wide significant variants for decline in FEV_1_, FVC, and FEV_1_/FVC, most of which represent novel signals for lung function decline. Our results, which overlapped with previously reported genetic signals for several related pulmonary traits, implicated 38 genes and were enriched for processes involving corticosteroid biosynthesis and metabolism, underscoring existing evidence for a role of corticosteroids in chronic lung health. Our findings contribute to our understanding of the genetics underlying age-related lung function decline and highlight relevant genes, biological pathways, and potential drug targets that could be repurposed to slow decline and treat lung disease.

## Supplementary Information

Supplementary Material 1.

Supplementary Material 2.

## Data Availability

Meta-analysis summary statistics are available from the corresponding author upon reasonable request. Preexisting data access policies for each contributing study specify that research data requests can be submitted to each steering committee; these will be promptly reviewed for confidentiality or intellectual property restrictions and will not unreasonably be refused.
