# Supplementary material for: Multi-ancestry genome-wide association study and meta-analysis of lung function decline
Source: Respir Res. 2026 Feb 28;27:110. doi: 10.1186/s12931-026-03565-x (PMC12961773; doi:10.1186/s12931-026-03565-x)
Supplement: Supplementary file 2 — Supplementary Material 2. [file 12931_2026_3565_MOESM2_ESM.docx]

Multi-ancestry genome-wide association study reveals novel genetic signals for lung function decline

SUPPLEMENTAL MATERIALS

# Study populations and spirometry measurements

*Atherosclerosis Risk in Communities (ARIC)*: The ARIC study is a population-based longitudinal cohort of about 15,800 primarily Black and White middle-aged men and women, recruited from four communities in the U.S. between 1987 and 1989. Spirometry was performed at clinic visits 1 (1987–1989), 2 (1990–1992), and 5 (2011–2013). Collins Survey II water-sealed spirometers (Warren E. Collins Inc., Braintree, MA) were used for visits 1 and 2, and SensorMedics model 1022 dry rolling seal spirometers (OMI, Houston, TX) were used for visit 5.^1^

*Coronary Artery Risk Development in Young Adults (CARDIA):* The CARDIA study is a population-based longitudinal cohort of about 5,115 Black and White young adults aged 18 to 30, recruited from four U.S. metropolitan areas: Birmingham, AL, Chicago, IL, Minneapolis, MN, and Oakland, CA. CARDIA began in 1985–1986.^1^ Spirometry was performed at baseline (1985–1986) and exam years 2 (1987–1988), 5 (1990–1991), 10 (1995–1996) and 20 (2005–2006). Collins Survey II water-sealed spirometers were used at baseline and exam years 2, 5 and 10, and SensorMedics model 1022 dry rolling seal spirometers were used at exam year 20.^1^

*Cardiovascular Health Study (CHS)***:**  CHS is a population-based longitudinal study of 520 men and women ≥ 65 years of age, recruited from four U.S. communities: Forsyth County, NC, Sacramento County, CA, Washington County, MD, and Allegheny County (Pittsburgh), PA, in 1989. An additional 687 Black men and women were recruited starting in 1992. Spirometry was performed at cohort examination 2 (1989–1990), 6 (1993–1994), 9 (1996–1997) and 18 (2005–2006). Collins Survey II water-sealed spirometers were used at exams 2, 6, and 9, and EasyOne flow-sensing spirometers (ndd Medical Technologies, Inc., Andover, MA) were used for exam 18.^1^ For this study, the exam 9 data for FVC were excluded due to concerns regarding comparability between the FVC measures at exams 6 and 9, as recommended by CHS investigators.^2^

*Framingham Heart Study (FHS)*: FHS is a household based, generational cohort in Framingham, MA, established in 1948. The Offspring cohort began in 1971 and is comprised of around 5,100 children of the original cohort and spouses of these children. The mean age at the time of enrollment was 36 years. Overall, 99.7% of all FHS participants (three generations) were self-reported White. Spirometry was performed at Offspring exams 3 (1983–1987), 5 (1991–1995), 6 (1995–1998), 7 (1998–2001), 8 (2005–2008), and 9 (2011–2014). Collins Survey II water-sealed spirometers were used for all exams.

*Health, Aging and Body Composition (HABC):* HABC is a population-based longitudinal study consisting of 3,075 Black and White men and women recruited from two US communities: Pittsburgh, PA and Memphis, TN, between March 1997 and July 1998. Participants were 70–79 years of age at recruitment. Spirometry was performed at baseline (1997–1998) and cohort examinations 5 (2001–2002, 8 (2004–2005), and 10 (2006–2007). SensorMedics model 1022 dry rolling seal spirometers were used for the baseline exam and exam 5, and EasyOne flow-sensing spirometers were used for exams 8 and 10.^1^

*Multi-Ethnic Study of Atherosclerosis (MESA):* MESA is a population-based cohort consisting of 6,814 Black, White, Hispanic, and Chinese-American men and women 45 to 84 years of age, recruited from six sites in the U.S., including St. Paul, MN, Los Angeles, CA, northern Manhattan, NY, Forsyth County, NC, Chicago, IL, and Baltimore City and County, MD, from July 2000 to August 2002. Spirometry was performed at examination 3 or 4 (2004–2007), 5 (2010–2011), and 6 (2016–2017). SensorMedics model 1022 dry rolling seal spirometers were used for all exams.^1^

*UK Biobank (UKBB):* The UKBB is a large-scale prospective study consisting of a half million UK residents, aged 40-69 at recruitment. Spirometry was performed in all participants at the initial assessment (2006–2010), and in a proportion of participants who were invited back for repeat assessments at three later time points (2012–2013, 2014+, and 2019+). Vitalograph Pneumotrac 6800 spirometers (Vitalograph Ltd., Buckingham, England) were used for all spirometry measurements.

*Evaluation of COPD Longitudinally to Identify Predictive Surrogate End-points (ECLIPSE)***:** The ECLIPSE study is a longitudinal observational study consisting of 2,164 smokers with COPD at the study baseline and a smaller number of smoking (337) and nonsmoking (245) controls without COPD at the study baseline. Recruitment occurred from December 2005-December 2006. Participants were aged 45-70 at recruitment and all participants were self-reported non-Hispanic White. Inclusion criteria for COPD cases included Global Initiative for Chronic Obstructive Lung Disease (GOLD) grades 2-4 by spirometry with post-bronchodilator FEV1 < 80% predicted and FEV1/FVC ≤ 0.7 and at least 10 pack-years of cigarette smoking. Inclusion criteria for controls included normal spirometry with post-bronchodilator FEV1>85% predicted and FEV1/FVC > 0.7. Spirometry was performed at study baseline, three months, six months and every six months thereafter for three years.^3,4^

*Genetic Epidemiology of Chronic Obstructive Pulmonary Disease (COPDGene)***:** The COPDGene study is an ongoing prospective observational study consisting of more than 10,000 smokers with and without COPD and a smaller number of non-smoking controls recruited from 21 clinical sites across the United States. Inclusion criteria included at least 10 pack-years of cigarette smoking (except for non-smoking controls) and self-identification as non-Hispanic white or African American, with planned enrollment of 2/3 non-Hispanic White and 1/3 African American. Spirometry was performed at the study baseline and the 5 and 10 year follow-up visits using the ndd EasyOne Spirometer (Zurich, Switzerland).^5,6^

Genotype, imputation and PC adjustment details for each study

| Cohort | Ancestry groups | Genotyping platform | Imputation server and reported values | QC steps and specific thresholds used for excluding genotyped variants | Genotype PC adjustment |
| --- | --- | --- | --- | --- | --- |
| ARIC | EA, AA | Affymetrix 6.0 | TOPMed, R^2^ | call rate<95%, HWE P<10^6^, MAF<1%, or no chromosomal location | 10 (ancestry specific) |
| CARDIA | EA, AA | Affymetrix 6.0 | TOPMed, R^2^ | call rate < 95%, HWE P < 10-5, duplicates, monomorphic variants, MAF < 1% (AA only) | 4 (ancestry specific) |
| CHS | EA, AA | Illumina 370CNV BeadChip (EA), Illumina HumanOmni1-Quad_v1 BeadChip (AA) | TOPMed, R^2^ | Call rate <97%, HWE P < 10-5, >2 duplicate errors or Mendelian inconsistencies. *At the time of analysis, variants were excluded for variance on the allele dosage <=0.01. | 6 (ancestry specific) |
| FHS | EA | Affymetrix 500K + 50K Human Gene Focused Panel | TOPMed, R^2^ | call rate < 96.9%, HWE P < 10-6, MAF < 1%, Mendelian errors > 1000, not being on chromosomes 1–22 or X, duplicates | 4 (EA specific) |
| HABC | EA, AA | Illumina Human1M-Duo | TOPMed, R^2^ | call rate < 95%, HWE P < 10^-6^, or MAF < 1% | 5 (ancestry specific) |
| MESA | EA, AA, HA, CHN | Affymetrix 6.0 | TOPMed, R^2^ | call rate < 95%, heterozygosity >53%, or monomorphic variants | 2 for EA, 1 for AA, 1 for CHN, 3 for HA |
| UKBB | EA | Affymetrix UK Biobank or UK BiLEVE Axiom Arrays | HRC, info score |  | 10 (EA specific) |

# Statistical analysis

Associations of genetic variants with lung function decline were evaluated in each cohort using general estimating equations with robust standard error and unstructured correlation structure. Repeated measurements of FEV_1_, FVC, or FEV_1_/FVC were regressed on variant, elapsed time since first lung function measurement, and the variant × elapsed time multiplicative interaction term. We focused on the variant x time interaction term to identify variants associated with lung function decline. The GEE model was selected based on its robustness for testing interaction terms in genetic association studies even when the interaction variable is mis-specified.^7^

Analyses were stratified by sex and self-reported race/ethnicity (as a proxy for genetic ancestry) and adjusted for covariates selected *a priori* based on prior knowledge of lung function predictors. These included baseline age, mean centered baseline age^2^, time-varying height, mean centered height^2^, weight (FVC only), baseline smoking status (current, former, never), current smoking at each spirometry measurement (yes/no), baseline smoking pack-years, cigarettes per day at each spirometry visit, study site (for multi-site cohorts), and genotype principal components. Mean centering of squared terms was done to minimize collinearity. Weight was included in the FVC model only based on its stronger association with a restrictive (FVC) versus obstructive (FEV_1_) phenotype. For cohorts that had a change in spirometry equipment during follow-up we also included a variable indicating whether lung function measurements occurred before or after the spirometer change. To remove extreme values driven by small sample sizes we filtered sex- and ancestry-specific results from each cohort to exclude variants present in <30 participants, variants with minor allele counts <20, and variants with effect sizes <−50 or >50 mL/year.

Replication of lung function decline-associated variants in COPD-enriched populations

Following the same analysis plan described for the discovery analyses, we tested decline-associated variants for replication in White (N=4,778) and Black (N=1,118) participants in two cohort studies enriched for COPD: the Genetic Epidemiology of COPD (COPDGene)^18^ and the Evaluation of COPD Longitudinally to Identify Predictive Surrogate End-points (ECLIPSE).^19^  Only variants passing QC filtering criteria (MAC>20 in at least two sex and ancestry strata) with genomic position and reference/alternate alleles matching those identified in the discovery analyses were tested. We used Bonferroni correction for the number of variants tested for assessing stringent statistical significance, along with a nominal significance threshold of p<0.05 for declaring suggestive replication. Because prevalent COPD and COPD severity may influence longitudinal lung function, we repeated the analyses adjusting for COPD status and disease stage (no COPD/preserved ratio impaired spirometry [PRISm]/GOLD status 1/2/3/4).^8–10^

### Heritability and genetic correlation with relevant pulmonary traits

Heritability of lung function decline and genetic correlation of decline phenotypes with relevant pulmonary traits were estimated in the FHS cohort (N=3,571 EA participants) using SOLAR. We limited this analysis to FHS for three reasons: (1) FHS is family-based so we could use family relationships to estimate heritability, (2) FHS is an optimal cohort for heritability calculations because of the number and time separation in the set of repeated measurements of lung function, and (3) we had access to individual-level data in FHS which was critical because our GWAS evaluated lung function decline with an interaction term and to our knowledge there are currently no validated methods for estimating heritability of interaction terms that use summary statistics. We evaluated decline phenotypes with evidence of significant heritability (p<0.05) for genetic correlation with baseline FEV_1_ and FVC, COPD at baseline, COPD at last observation, carbon monoxide diffusion capacity (DLCO), asthma, immunoglobulin E (IgE), emphysema, and interstitial lung abnormality (ILA). All heritability and genetic correlation analyses accounted for sex, baseline age, and ever/never smoking status.

### Gene-based tests and functional validation analyses

Gene-based tests were performed in MAGMA for each set of meta-analysis results using the SNP-wise mean, SNP-wise top, and SNP-wise multi (aggregate of SNP-wise mean and SNP-wise top) methods.^11^ Genes with z-scores significant at Bonferroni-corrected p-value thresholds (p<0.05/18,156 genes) for one or more of the MAGMA methods were considered significant. Gene expression in lung tissue was evaluated using the GTEx platform.^12^ We further evaluated decline-associated genes for associations with relevant pulmonary traits reported in the GWAS catalogue and Phenoscanner databases using the LDlinkR and Phenoscanner R packages.^13–17^

Colocalization of decline-associated variants with expression quantitative trait loci (eQTL) was performed using TOPMed Freeze 2.RNA cis-eQTL results for whole blood (N=6,454, MAF cutoff=0.1%) and lung tissue (N=1,291, MAF cutoff=1%).^18^ The TOPMed Freeze 2.RNA eQTL results were generated in a collaboration between the TOPMed Informatics Research Center, TOPMed Multi-Omics working group, and the TOPMed parent studies contributing RNA-seq, and distributed to TOPMed investigators. The whole blood cis-eQTLs represent a genetically diverse population (based on genetically inferred global ancestry, 50.8% of whole blood samples were >= 90% EUR, while 46.7% were admixed, mostly representing EUR, AFR, and AMR ancestry). The lung cis-eQTLs represent a predominantly European ancestry population (87.0% of lung samples were >=90% EUR while 11.4% of lung samples were admixed). Colocalization analyses were performed separately for decline-associated variants from each set of meta-analysis results, using GRCh38 defined regions of +/- 500kb around each of the 361 decline-associated variants identified in our GWAS analyses. All colocalization analyses were run with the R package coloc using the coloc.abf function with default settings.^19^ Evidence for colocalization was determined by the region-level posterior probability for the hypothesis that both traits share the same causal variant (ppH4). We considered ppH4 >0.8 to be strong evidence for colocalization, and ppH4 >0.5 to be moderate evidence for colocalization. Causal variants for regions with ppH4>0.5 were further evaluated through SNP level results and locus plots.

We identified genetically-predicted protein level associations with lung function decline phenotypes within the established S-PrediXcan framework.^20^ S-PrediXcan integrates GWAS summary statistics with protein prediction models and colocalization to infer protein-trait associations without requiring individual-level genetic data. This approach of using GWAS summary statistics allows for inference on a large sample size without having to perform a pooled analysis of individual level data. The protein prediction models are pQTL-based elastic net models derived from European and African ancestry individuals from the Atherosclerosis Risk in Communities (ARIC) cohort.^20–22^ S-PrediXcan analyses were run separately for each GWAS meta-analysis result. Ancestry-matched pQTLs were used for the ancestry-specific meta-analyses. European ancestry pQTLs were used for the cross-ancestry meta-analyses because cross-ancestry pQTLs were not available and the majority of participants in the cross-ancestry meta-analysis were of European ancestry. Protein associations with Benjamini-Hochberg false discovery rate adjusted p-values < 0.1 were considered significant.

Enrichment analyses were based on FUMA-based positional mapping of candidate variants. We evaluated the enrichment of pre-defined gene sets obtained from the Molecular Signatures Database, WikiPathways and reported genes from the GWAS catalogue with the FUMA GENE2FUNC function.^23^ Multiple testing correction was performed separately for each gene-set category. Gene sets with Benjamini-Hochberg false discovery rate adjusted p-values <0.05 were considered enriched. Pathway and disease enrichment patterns were explored using the Genomic Regions Enrichment of Annotations Tool (GREAT).^24,25^ Pathway and disease terms with false discovery rate–adjusted p-values <0.05 for binomial or hypergeometric tests were declared significant.

**Acknowledgements for TOPMed e/sQTL results:**

Freeze 2.RNA TOPMed e/sQTL results were generated in a collaboration between the TOPMed Informatics Research Center, TOPMed Multi-Omics working group, and the TOPMed parent studies contributing RNA-seq and distributed to TOPMed investigators. We acknowledge the contributing cohorts, sequencing centers, and the TOPMed IRC.

Molecular data for the Trans-Omics in Precision Medicine (TOPMed) program was supported by the National Heart, Lung and Blood Institute (NHLBI).

Genome Sequencing and Related Phenotypes in the Framingham Heart Study” (phs000974)” was performed at the Northwest Genomics Center (HHSN268201600032I). Genome Sequencing for “NHLBI TOPMed: Whole Genome Sequencing and Related Phenotypes in the Framingham Heart Study” (phs000974)” was performed at Broad Genomics (HHSN268201600034I, 3U54HG003067-12S2, 3R01HL092577-06S1). RNASeq for “NHLBI TOPMed: Genetic Epidemiology of COPD (COPDGene) (phs000951)” was performed at the Northwest Genomics Center (HHSN268201600032I). Genome Sequencing for “NHLBI TOPMed: Genetic Epidemiology of COPD (COPDGene) (phs000951)” was performed at Broad Genomics (HHSN268201500014C) and the Northwest Genomics Center (3R01HL089856-08S1). RNASeq for “NHLBI TOPMed - NHGRI CCDG: Genes-Environments and Admixture in Latino Asthmatics (GALA II) (phs000920)” was performed at Broad Genomics (HHSN268201600034I). Genome Sequencing for ““NHLBI TOPMed - NHGRI CCDG: Genes-Environments and Admixture in Latino Asthmatics (GALA II) (phs000920)” was performed at NYGC Genomics (3R01HL117004-02S3). RNASeq for “NHLBI TOPMed: Study of African Americans, Asthma, Genes and Environment (SAGE) (phs000921)” was performed at Broad Genomics (HHSN268201600034I). Genome Sequencing for “NHLBI TOPMed: Study of African Americans, Asthma, Genes and Environment (SAGE) (phs000921)” was performed at NYGC Genomics (3R01HL117004-02S3) and the Northwest Genomics Center (HHSN268201600032I). RNASeq for “NHLBI TOPMed: SubPopulations and InteRmediate Outcome Measures In COPD Study (SPIROMICS) (phs001927)” was performed at Northwest Genomics Center (HHSN268201600032I). Genome Sequencing for “NHLBI TOPMed: SubPopulations and InteRmediate Outcome Measures In COPD Study (SPIROMICS) (phs001927)” was performed at Broad Genomics (HHSN268201600034I). RNASeq for “NHLBI TOPMed: MESA and MESA Family AA-CAC (phs001416)” was performed at Northwest Genomics Center (HHSN268201600032I) and Broad Genomics (HHSN268201600034I). Genome Sequencing for “NHLBI TOPMed: MESA and MESA Family AA-CAC (phs001416)” was performed at Broad Genomics (3U54HG003067-13S1, HHSN268201600034I, HHSN268201500014C). RNASeq for “NHLBI TOPMed: Women's Health Initiative (WHI) (phs001237)” was performed at Broad Genomics (HHSN268201600034I). Genome Sequencing for “NHLBI TOPMed: Women's Health Initiative (WHI) (phs001237)” was performed at Broad Genomics (HHSN268201500014C). RNASeq for “NHLBI TOPMed: Lung Tissue Research Consortium (LTRC) (phs001662)” was performed at Northwest Genomics Center (HHSN268201600032I). RNASeq for “NHLBI TOPMed: Lung Tissue Research Consortium (LTRC) (phs001662)” was performed at Broad Genomics (HHSN268201600034I). Core support including centralized genomic read mapping and genotype calling, along with variant quality metrics and filtering were provided by the TOPMed Informatics Research Center (3R01HL-117626-02S1; contract HHSN268201800002I). Core support including phenotype harmonization, data management, sample-identity QC, and general program coordination were provided by the TOPMed Data Coordinating Center (R01HL-120393; U01HL-120393; contract HHSN268201800001I). We gratefully acknowledge the studies and participants who provided biological samples and data for TOPMed.

**REFERENCES**

1. Oelsner, E.C., Balte, P.P., Cassano, P.A., Couper, D., Enright, P.L., Folsom, A.R., Hankinson, J., Jacobs, D.R., Kalhan, R., Kaplan, R., et al. Harmonization of Respiratory Data From 9 US Population-Based CohortsThe NHLBI Pooled Cohorts Study. Am. J. Epidemiol. https://doi.org/10.1093/aje/kwy139.

2. Spirometry Comparability | chs-nhlbi https://chs-nhlbi.org/internal/SpirometryComparability.

3. Hurst, J.R., Vestbo, J., Anzueto, A., Locantore, N., Müllerova, H., Tal-Singer, R., Miller, B., Lomas, D.A., Agusti, A., MacNee, W., et al. (2010). Susceptibility to Exacerbation in Chronic Obstructive Pulmonary Disease. N. Engl. J. Med. *363*, 1128–1138. https://doi.org/10.1056/NEJMoa0909883.

4. Vestbo, J., Anderson, W., Coxson, H.O., Crim, C., Dawber, F., Edwards, L., Hagan, G., Knobil, K., Lomas, D.A., MacNee, W., et al. (2008). Evaluation of COPD Longitudinally to Identify Predictive Surrogate End-points (ECLIPSE). Eur. Respir. J. *31*, 869–873. https://doi.org/10.1183/09031936.00111707.

5. Regan, E.A., Hokanson, J.E., Murphy, J.R., Make, B., Lynch, D.A., Beaty, T.H., Curran-Everett, D., Silverman, E.K., and Crapo, J.D. (2010). Genetic Epidemiology of COPD (COPDGene) Study Design. COPD *7*, 32–43. https://doi.org/10.3109/15412550903499522.

6. Maselli, D.J., Bhatt, S.P., Anzueto, A., Bowler, R.P., DeMeo, D.L., Diaz, A.A., Dransfield, M.T., Fawzy, A., Foreman, M.G., Hanania, N.A., et al. (2019). Clinical Epidemiology of COPD: Insights From 10 Years of the COPDGene Study. Chest *156*, 228–238. https://doi.org/10.1016/j.chest.2019.04.135.

7. Tchetgen Tchetgen, E.J., and Kraft, P. (2011). On the robustness of tests of genetic associations incorporating gene-environment interaction when the environmental exposure is mis-specified. Epidemiol. Camb. Mass *22*, 257–261. https://doi.org/10.1097/EDE.0b013e31820877c5.

8. Wan, E.S., Fortis, S., Regan, E.A., Hokanson, J., Han, M.K., Casaburi, R., Make, B.J., Crapo, J.D., DeMeo, D.L., Silverman, E.K., et al. (2018). Longitudinal Phenotypes and Mortality in Preserved Ratio Impaired Spirometry in the COPDGene Study. Am. J. Respir. Crit. Care Med. *198*, 1397–1405. https://doi.org/10.1164/rccm.201804-0663OC.

9. Tantucci, C., and Modina, D. (2012). Lung function decline in COPD. Int. J. Chron. Obstruct. Pulmon. Dis. *7*, 95–99. https://doi.org/10.2147/COPD.S27480.

10. Kim, J., Yoon, H.I., Oh, Y.-M., Lim, S.Y., Lee, J.-H., Kim, T.-H., Lee, S.Y., Lee, J.H., Lee, S.-D., and Lee, C.-H. (2015). Lung function decline rates according to GOLD group in patients with chronic obstructive pulmonary disease. Int. J. Chron. Obstruct. Pulmon. Dis. *10*, 1819. https://doi.org/10.2147/COPD.S87766.

11. de Leeuw, C.A., Mooij, J.M., Heskes, T., and Posthuma, D. (2015). MAGMA: Generalized Gene-Set Analysis of GWAS Data. PLoS Comput. Biol. *11*, e1004219. https://doi.org/10.1371/journal.pcbi.1004219.

12. Lonsdale, J., Thomas, J., Salvatore, M., Phillips, R., Lo, E., Shad, S., Hasz, R., Walters, G., Garcia, F., Young, N., et al. (2013). The Genotype-Tissue Expression (GTEx) project. Nat. Genet. *45*, 580–585. https://doi.org/10.1038/ng.2653.

13. GWAS Catalog https://www.ebi.ac.uk/gwas/.

14. Staley, J.R., Blackshaw, J., Kamat, M.A., Ellis, S., Surendran, P., Sun, B.B., Paul, D.S., Freitag, D., Burgess, S., Danesh, J., et al. (2016). PhenoScanner: a database of human genotype-phenotype associations. Bioinforma. Oxf. Engl. *32*, 3207–3209. https://doi.org/10.1093/bioinformatics/btw373.

15. Kamat, M.A., Blackshaw, J.A., Young, R., Surendran, P., Burgess, S., Danesh, J., Butterworth, A.S., and Staley, J.R. (2019). PhenoScanner V2: an expanded tool for searching human genotype–phenotype associations. Bioinformatics *35*, 4851–4853. https://doi.org/10.1093/bioinformatics/btz469.

16. Myers, T.A., Chanock, S.J., and Machiela, M.J. (2020). LDlinkR: An R Package for Rapidly Calculating Linkage Disequilibrium Statistics in Diverse Populations. Front. Genet. *11*, 157. https://doi.org/10.3389/fgene.2020.00157.

17. R: PhenoScanner https://search.r-project.org/CRAN/refmans/MendelianRandomization/html/phenoscanner.html.

18. Orchard, P., Blackwell, T.W., Kachuri, L., Castaldi, P.J., Cho, M.H., Christenson, S.A., Durda, P., Gabriel, S., Hersh, C.P., Huntsman, S., et al. (2025). Cross-cohort analysis of expression and splicing quantitative trait loci in TOPMed. Preprint at medRxiv, https://doi.org/10.1101/2025.02.19.25322561 https://doi.org/10.1101/2025.02.19.25322561.

19. Rasooly, D., Peloso, G.M., and Giambartolomei, C. (2022). Bayesian Genetic Colocalization Test of Two Traits Using coloc. Curr. Protoc. *2*, e627. https://doi.org/10.1002/cpz1.627.

20. Barbeira, A.N., Dickinson, S.P., Bonazzola, R., Zheng, J., Wheeler, H.E., Torres, J.M., Torstenson, E.S., Shah, K.P., Garcia, T., Edwards, T.L., et al. (2018). Exploring the phenotypic consequences of tissue specific gene expression variation inferred from GWAS summary statistics. Nat. Commun. *9*, 1825. https://doi.org/10.1038/s41467-018-03621-1.

21. Mi, S. (2022). Protein prediction models - ARIC | PredictDB. https://predictdb.org/post/2022/11/14/protein-prediction-models/.

22. Zhang, J., Dutta, D., Köttgen, A., Tin, A., Schlosser, P., Grams, M.E., Harvey, B., Yu, B., Boerwinkle, E., Coresh, J., et al. (2022). Plasma proteome analyses in individuals of European and African ancestry identify cis-pQTLs and models for proteome-wide association studies. Nat. Genet. *54*, 593–602. https://doi.org/10.1038/s41588-022-01051-w.

23. Watanabe, K., Taskesen, E., van Bochoven, A., and Posthuma, D. (2017). Functional mapping and annotation of genetic associations with FUMA. Nat. Commun. *8*, 1826. https://doi.org/10.1038/s41467-017-01261-5.

24. McLean, C.Y., Bristor, D., Hiller, M., Clarke, S.L., Schaar, B.T., Lowe, C.B., Wenger, A.M., and Bejerano, G. (2010). GREAT improves functional interpretation of cis-regulatory regions. Nat. Biotechnol. *28*, 495–501. https://doi.org/10.1038/nbt.1630.

25. Tanigawa, Y., Dyer, E.S., and Bejerano, G. (2022). WhichTF is functionally important in your open chromatin data? PLoS Comput. Biol. *18*, e1010378. https://doi.org/10.1371/journal.pcbi.1010378.

Figure S1) Quantile-quantile plots and genomic inflation values for cross-ancestry and ancestry-specific meta-analyses. The plots compare the observed vs. expected *P* values for testing of the variant by time interaction term in relation to FEV_1_, FVC or FEV_1_/FVC. The corresponding genomic inflation factors are shown, as calculated across all variants before the exclusion of previously implicated variants.

Figure S2) Violin plot of minor allele frequency (MAF) distributions of variants with p<5E-08 across decline phenotypes and ancestry. The plot shows density curves for the log_10_ transformed MAF distribution of significant variants identified from the cross-ancestry and ancestry specific analyses for decline in FEV_1_, FVC and FEV_1_/FVC. The dotted red line represents MAF of 0.05.

Figure S3) Circular Manhattan plot for European Ancestry analysis. Genome-wide results for decline in FEV_1_ (outer circle), FVC (middle circle), and FEV_1_/FVC (inner circle) from the European ancestry analyses. Dotted red lines denote the genome-wide significance threshold of p = 5E-08.  Red circles represent variants passing genome-wide significance.


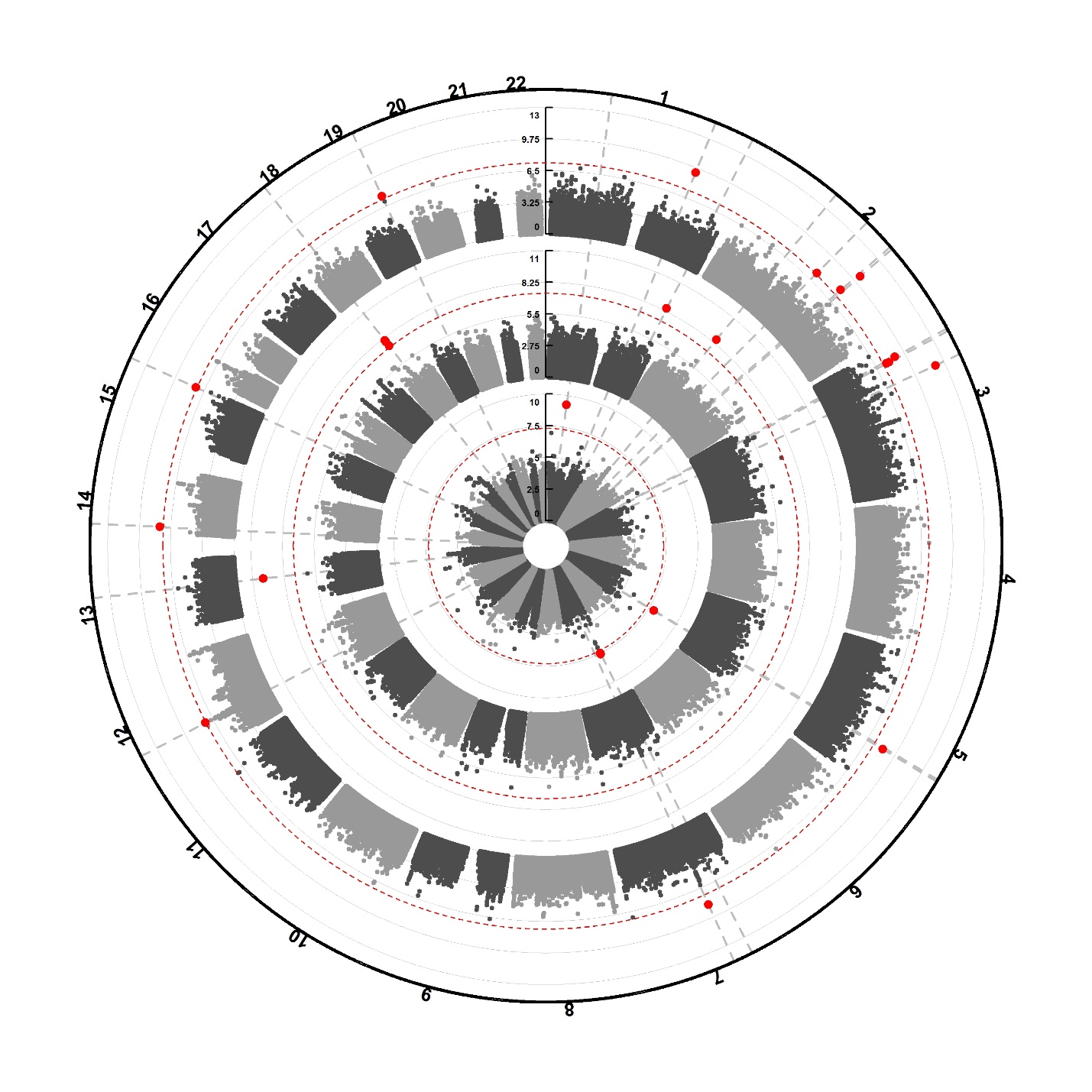


**FEV_1_/FVC**

**FVC**

**FEV1**

Figure S4) Circular Manhattan plot for African ancestry analyses. Genome-wide results for decline in FEV_1_ (outer circle), FVC (middle circle), and FEV_1_/FVC (inner circle) from the African ancestry analyses. Dotted red lines denote the genome-wide significance threshold of p = 5E-08.  Red circles represent variants passing genome-wide significance.


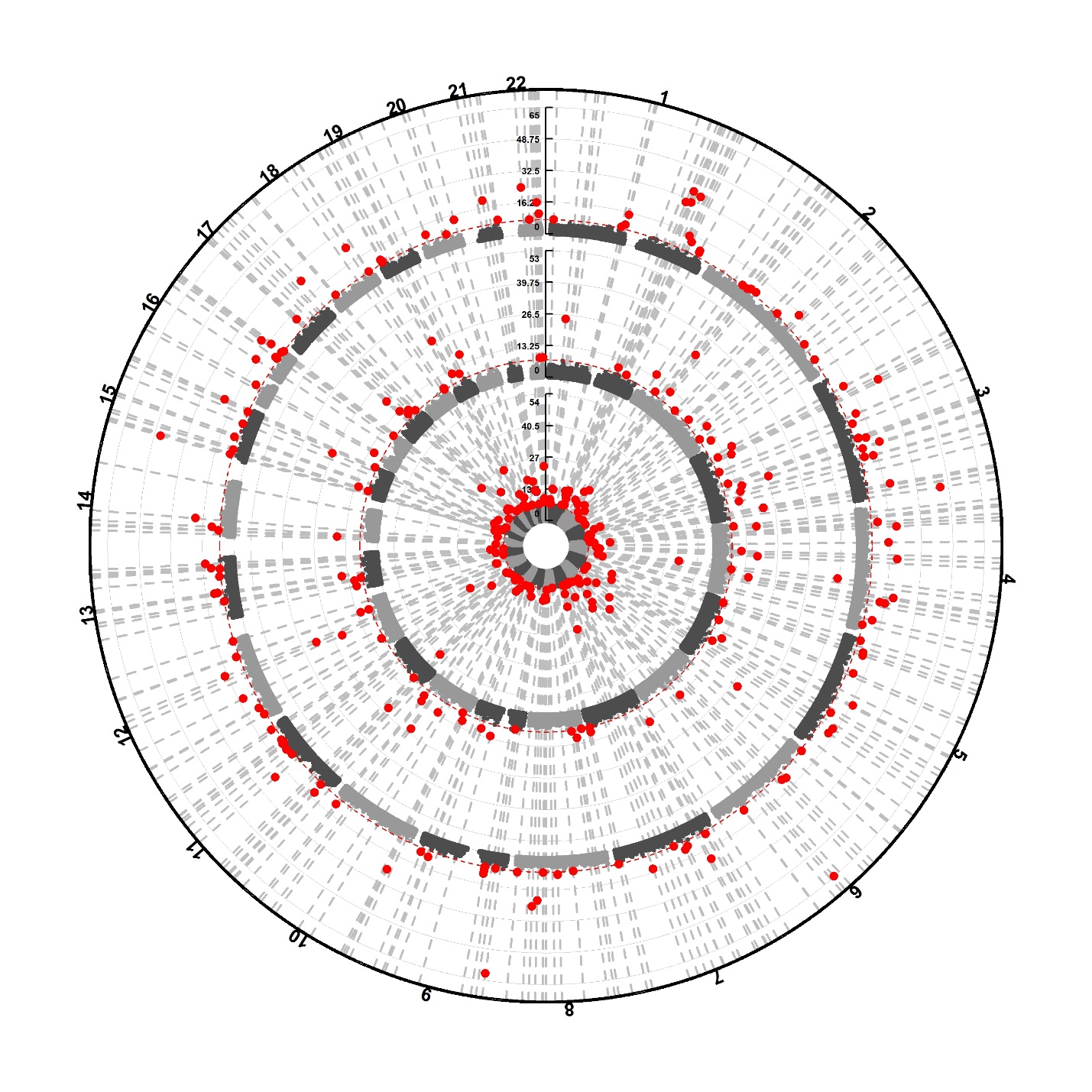


**FEV_1_**

**FEV_1_/FVC**

**FVC**

**Figure S5) Locus plots of regions with eQTLs distinct from GWAS signals.** Locus plots overlaying our GWAS results (blue circles) with the TOPMed blood or lung eQTL results (orange triangles) for the regions with suggestive evidence of colocalization (region ppH4>0.5). Plots show the +/-500kb around a) rs143624225 (chr7) for FEV1 decline in AA and blood eQTL and b) rs79222894 (chr9) for ratio decline in AA and lung eQTL, and c) rs115482155 (chr 18) for ratio decline in AA and lung eQTL. The variants with the largest SNP-level ppH4 are enlarged, highlighting that the eQTLs in these regions are unlikely to colocalize with the decline-associated variants. GRCh38 genomic positions are displayed.

a)

Genomic position (GRCh38): Chr7

rs143624225

Genomic position (GRCh38): Chr9

rs79222894

b)

c)

Genomic position (GRCh38): Chr18

rs115482155

**Figure S6) Heatmap of S-PrediXcan predicted protein associations with decline phenotypes.** Z scores of S-PrediXcan results for all proteins associated at nominal significance (p<0.05) with at least one decline phenotype are shown. Red indicates positive Z-scores and blue indicates negative z-scores. ***denotes associations significant at FDR<0.05, *denotes associations significant at the nominal threshold of 0.05. Light grey shading indicates that a protein was not represented in a given S-PrediXcan result.
